# Pancreaticobiliary maljunction and pancreas divisum accompanied with intestinal malrotation: a case report

**DOI:** 10.1186/s12887-022-03171-y

**Published:** 2022-02-28

**Authors:** Waiun Lei, Jiayu Yan, Tingchong Zhang, Lu Liu, Yajun Chen

**Affiliations:** Department of General Surgery, Beijing Children’s Hospital, National Center for Children’s Health, Capital Medical University, No. 56 Nalishi Road, Xicheng District, CN 100045 Beijing, China

**Keywords:** Pancreaticobiliary maljunction, Intestinal malrotation, Pancreas divisum, Laparoscopy, Roux-en-Y hepaticojejunostomy, Ladd’s procedure, Case report

## Abstract

**Background:**

Pancreaticobiliary maljunction is a congenital anatomical abnorma l junction of the pancreatic duct and bile duct into a common channel outside the duodenal wall. Pancreas divisum is also a congenital anatomical abnormality characterized by unfused pancreatic ducts. Intestinal malrotation is caused by the failure of bowel rotation and fixation. We reported an optimal surgical intervention for the rare case of pancreaticobiliary maljunction and pancreas divisum accompanied intestinal malrotation.

**Case presentation:**

A 2-year-old female presented with fever and jaundice. Abdominal ultrasound showed dilated common bile duct and intrahepatic bile ducts; MRCP showed pancreaticobiliary maljunction, pancreas divisum, and dilated biliary system; Abdominal contrast-enhanced CT showed a reversed relationship between the superior mesenteric artery and the superior mesenteric vein. An operation of laparoscopic resection of the extrahepatic bile duct, Roux-en-Y hepaticojejunostomy, and Ladd’s procedure was performed after the inflammation of the biliary system was treated. The post-operative follow-up period was uneventful.

**Conclusions:**

The management of pancreas divisum can be conservative. We present an optimal pattern of Roux-en-Y hepaticojejunostomy to deal with pancreaticobiliary maljunction associated with intestinal malrotation.

## Background

Pancreaticobiliary maljunction (PBM) is a congenital anatomical abnormal junction of the pancreatic duct and bile duct into a common channel outside the duodenal wall. Cholangitis, biliary stones, pancreatitis may occur due to the regurgitation of pancreatic juice into the biliary system and bile into the pancreatic duct. Resection of the extrahepatic bile duct and Roux-en-Y hepaticojejunostomy is regarded as the treatment of choice for PBM [1]. Pancreatic divisum is also a congenital anatomical abnormality of the pancreas, which is characterized by unfused pancreatic ducts. Most of the cases are asymptomatic. Acute pancreatitis, chronic pancreatitis or chronic abdominal pain are the main clinical symptoms and occur in 5% of the patients with pancreas divisum. The management of pancreas divisum can be conservative when no complications occur [2]. Intestinal malrotation is caused by failure of the bowel rotation and fixation. Patient can be asymptomatic. However, most cases present with bilious vomiting or nonspecific symptoms like intermittent vomiting, abdominal pain, and poor weight gain. Intestinal malrotation is typically treated using the Ladd’s procedure [3].

The co-occurrence of PBM and pancreatic divisum is uncommon. Endoscopic retrograde cholangiopancreatography (ERCP) can be performed to remove the biliary stones and place a biliary stent [4]. Cases of PBM (or choledochal cyst) coexisting with intestinal malrotation are rare, most of which have been reported in Japan [5]. Additionally, PBM and pancreas divisum coexisting with intestinal malrotation are extremely rare, the management of which can be challenging due to its rarity and complexity, and should be approached cautiously. In this article, a case of pancreaticobiliary maljunction associated with pancreas divisum and intestinal malrotation is reported and the optimal surgical intervention is presented.

## Case presentation

**Fig. 1:**
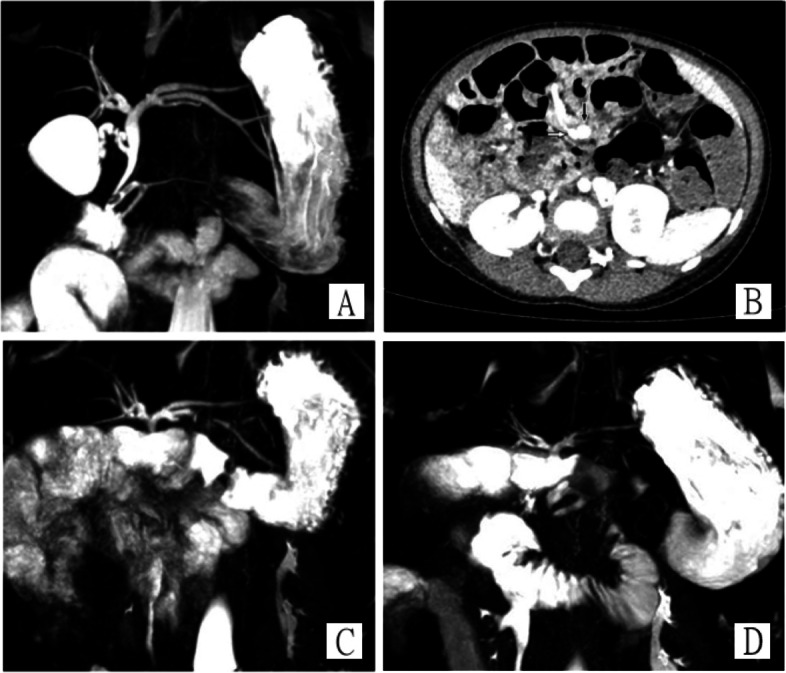
Imaging study on admission and post-operation. **A** MRCP showed dilated biliary system, a bile stone in the common bile duct, pancreaticobiliary maljunction, and pancreas divisum. **B** Abdominal CT contrast showed SMV (black arrow) positioning on the left of SMA (white arrow). **C** MRCP at 2 months after surgery showed the dilation of the intrahepatic bile duct is improved, the biliary-enteric anastomosis is unobstructed, no stricture, and no leak. **D** MRCP at 12 months after surgery

A 2-year-old female was referred to Beijing Children’s Hospital with chief complaints of fever and jaundice. The patient had experienced fever with chills for 7 days with a peak body temperature of 39.6 °C. Blood samples were taken at the local clinic, which demonstrated a significant elevation of C-reactive protein (CRP, 127.6 mg/L) and white blood cells count (WBC, 38.23 × 10^9/L). Physical examination was performed at our center, which showed remarkable tenderness at the right upper quadrant of the abdomen. Laboratory data from blood samples demonstrated the significant elevation of CRP (> 160 mg/L), WBC (52.73 × 10^9/L), ALT (86.9 U/L), AST (91.1 U/L), ALP (1116 U/L), γ-GT (1334.9 U/L), TBil (128.15 µmol/L), DBil (115.93 µmol/L), PT (15.3 S) and APTT (46.2 S) were prolonged, but serum ammonia and amylase were normal. Abdominal ultrasound showed dilation of the common bile duct and intrahepatic bile ducts, with a 0.6 × 0.2 cm bile stone in the common bile duct. MRCP showed pancreas divisum, dilated bile duct and pancreaticobiliary maljunction with the common channel located 1 cm away from the duodenal wall (Fig. 1 A). Abdominal contrast-enhanced CT showed a reversed relationship between the superior mesenteric artery and the superior mesenteric vein, which is a sign of intestinal malrotation (Fig. 1B). Ertapenem (INVANZ®) for biliary tract infections was administered intravenously for 2 weeks until the laboratory data were normal. Thereafter, laparoscopic resection of the extrahepatic bile duct, Roux-en-Y hepaticojejunostomy and Ladd’s procedure was scheduled.

**Fig. 2:**
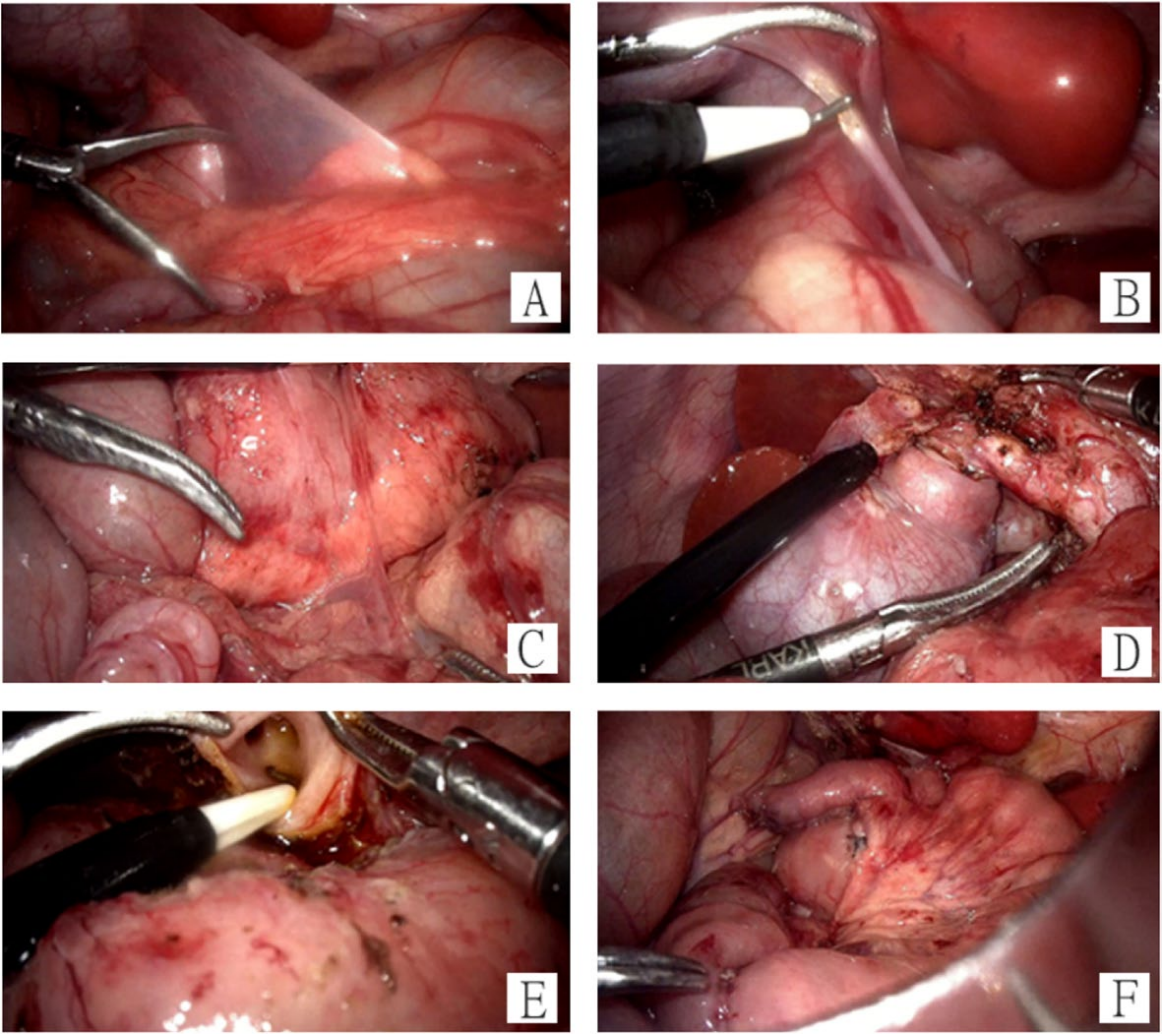
Intraoperative images. **A** The cecum is located into the right epigastrium, Ladd’s bands cross from the cecum to the duodenum and the gallbladder. **B** Lysis of Ladd’s bands. **C** Duodenum is 270 degrees surrounded by pancreatic tissue. **D** Fusiform dilation of the common bile duct. **E** The left and right hepatic duct openings were identified. **F** Reversed Roux-en-Y hepaticojejunostomy

Operative details: After general anesthesia was induced. In supine position, an umbilical cannula for a 3-dimension telescope was inserted, CO2 pneumoperitoneum pressure and flow rate were ranged from 10 to 12 mmHg and 2.0 to 2.5 L/min, after which three other trocars for instruments were inserted through the right hypochondrial region, right lumbar region at midclavicular line, and left lumbar region at midclavicular line of the abdomen. Inspection of the peritoneal cavity revealed that the duodenojejunal flexure lay to the right of the midline, the cecum was displaced from its usual position in the right lower quadrant to the right epigastrium, with peritoneal folds (Ladd’s bands) crossing from the cecum to duodenum and gallbladder (Fig. 2 A). A Ladd’s procedure (division of peritoneal bands and prophylactic appendectomy) was performed (Fig. 2B C). After lysing the Ladd’s bands, the fusiform dilation of the common bile duct was inspected. Resection of gallbladder and bile ducts was performed until the left and right hepatic duct openings were identified (Fig. 2D and E). The umbilical incision was then extended, and a reversed Roux-en-Y anastomosis was performed extra-abdominally, which was 15 cm away from the duodenojejunal flexure with a Roux limb of approximately 20 cm (Fig. 2 F). The bowel was returned to the peritoneal cavity through the umbilical incision, and pneumoperitoneum was reestablished. A hepaticojejunostomy was done laparoscopically, the defect of the mesentery was sutured. Last, a drainage tube was placed underneath the porta hepatis.

Cefoperazone/sulbactam (SULPERAZONE®) and metronidazole (FLAGYL®) were administered intravenously after the operation. The nasogastric tube was withdrawn 5 days after surgery, and the patient was allowed to drink water. The drainage tube was removed 1 week after the operation and a low-fat diet started. The patient was discharged 2 weeks after the operation with no symptoms or abnormalities in laboratory data. There were two follow-up visits 2 months and 12 months after the surgery. Complete blood count, serum biochemistry and coagulation profile remained normal. Abdominal ultrasonography showed no bowel obstruction, evidence of adhesions or volvulus of the midgut. MRCP showed no dilated intrahepatic bile ducts, obstructed biliary-enteric anastomosis or leaks (Fig. 1 C-D).

## Discussion and conclusion

PBM is a rare congenital anatomical abnormality with incidence in western countries about 1/100,000 individuals. However, the incidence is estimated to be 100–1000 times higher in Asian countries, especially in Japan [1]. Pancreas divisum, a congenital abnormality characterized by unfused pancreatic ducts, is associated with acute or chronic pancreatitis. However, most of the cases reported are asymptomatic [2]. Intestinal malrotation is caused by failure of the bowel rotation and fixation, affects about 1 in 5000 live births. Most cases present in the neonatal period, with bilious vomiting, while older children tend to present with nonspecific symptoms such as intermittent vomiting, abdominal pain, and limited weight gain. Occasionally, malrotation may be found on imaging studies incidentally [3].

**Fig. 3:**
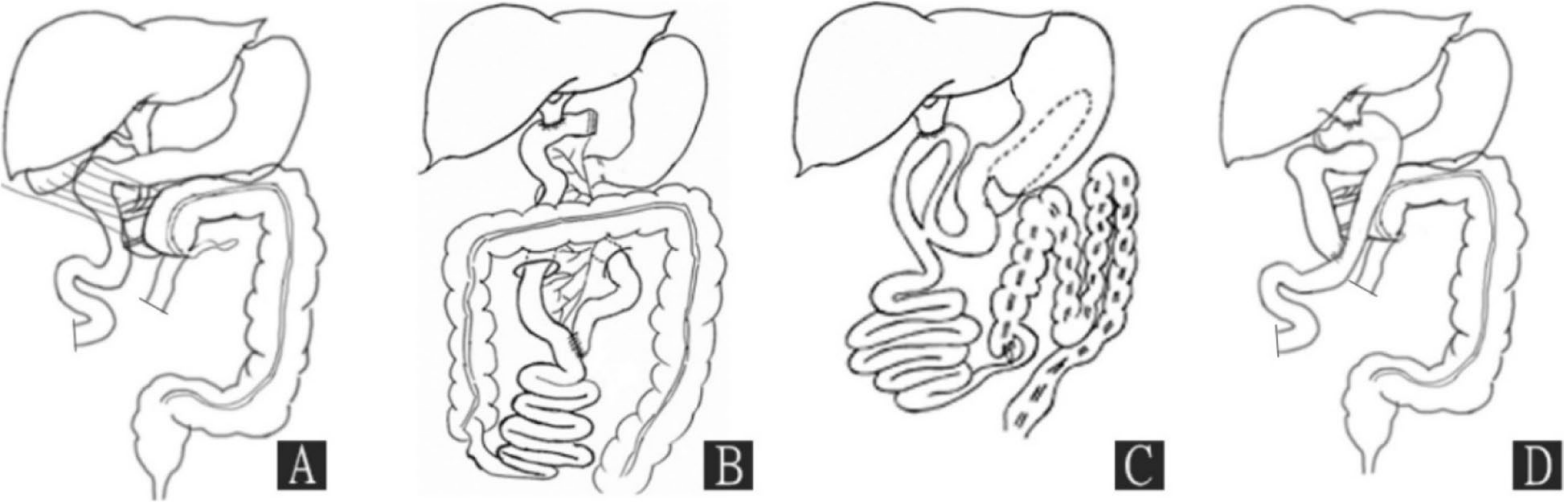
Anatomical illustrations. **A** Illustration of coexistence malformation in our case. **B** Routine Roux-en-Y hepaticojejunostomy. **C** Loop hepaticojejunostomy. **D** Illustration of the operation in our case: Lysis of Ladd’s bands, prophylactic appendectomy, resection of gallbladder and bile ducts, reverse Roux-en-Y hepaticojejunostomy

The development of the midgut and the biliary system both occur between the 5th to 10th week of gestation. Campbell et al. concluded that malrotation and biliary tract malformations can occur simultaneously [6]. However, intestinal malrotation should be independent of pancreatic division and PBM because while the fusion of ventral and dorsal pancreas occurs between the 5th to 10th week of gestation, the midgut rotation occurs between the 10th to 12th week of gestation [7]. The coexistence of PBM, intestinal malrotation, and pancreas divisum found in our case has rarely been reported in the literature (Fig. 3 A).

Laparoscopic resection of the extrahepatic bile duct and Roux-en-Y hepaticojejunostomy (Fig. 3B) is the treatment of choice for congenital biliary dilation (or choledochal cyst). In cases of choledochal cyst accompanied with intestinal malrotation, the approach of hepaticojejunostomy should be adjusted, because the anatomical position of the duodenum, the jejunum and the colon are significantly different from those patients without intestinal malrotation. Norihiro et al. reported a case of congenital biliary dilatation with un-fused pancreatic duct system and intestinal malrotation treated by open resection of the extrahepatic bile duct, hepaticojejunostomy and Ladd’s procedure (Fig. 3 C). The postoperative recovery was good [8]. Shirai et al. reported 3 cases of choledochal cyst with intestinal malrotation that were treated by open resection of the extrahepatic bile duct, hepaticoduodenostomy and Ladd’s procedure. Postoperative adhesive intestinal obstruction and duodenogastric reflux occurred in all 3 cases, and they ultimately converted to hepaticojejunostomy. After conversion, long-term follow-up was uneventful [5]. In our case, we designed a reverse Roux-en-Y hepaticojejunostomy (Fig. 3D). Compared with routine Roux-en-Y hepaticojejunostomy, the biliary-enteric anastomosis is smoother, and the possibility of compression or distortion is relatively minor. Moreover, the resection of the extrahepatic bile duct and the hepaticojejunostomy were performed laparoscopically, which is minimally invasive. We suggest that the pattern of hepaticojejunostomy should be designed according to the anatomical position of the intestine, following the fundamental principle that the alimentary tract should not be twisted or folded.

The combination of PBM, pancreas divisum, and intestinal malrotation is rare, therefore surgical intervention should be designed individually according to the patient’s anatomical abnormalities. We report a rare case of PBM and pancreas divisum accompanied with intestinal malrotation treated by laparoscopic Ladd’s procedure, resection of the extrahepatic bile duct and an optimal pattern of Roux-en-Y hepaticojejunostomy. We are of the opinion that this case adds to the literature and practice regarding hepatobiliary and gastrointestinal surgical management of similar rare cases.

## Data Availability

All data generated or analyzed during this study are included in this manuscript.

## Abbreviations

CT: Computed tomography
US: Ultrasound
MRI: Magnetic resonance imaging
MRCP: Magnetic resonance cholangiopancreatography
ERCP: Endoscopic retrograde cholangiopancreatography
PBM: Pancreaticobiliary maljunction
CBC: Complete blood count
CRP: C-reactive protein
WBC: White blood cells
N%: Neutrocyte%
PLT: Platelet
ALT: Alanine transaminase
AST: Aspartate transaminase
ALP: Alkaline phosphatase
γ-GT: Gamma-glutamyl transferase
TBil: Total bilirubin
DBil: Direct bilirubin
IBil: Indirect bilirubin
AMON: Ammonia
AMY: Amylase
PT: Prothrombin time
APTT (S): Activated partial thromboplastin time
INR: International normalized ratio
